# Simulating the Impact of the Natural Radiation Background on Bacterial Systems: Implications for Very Low Radiation Biological Experiments

**DOI:** 10.1371/journal.pone.0166364

**Published:** 2016-11-16

**Authors:** Nathanael Lampe, David G. Biron, Jeremy M. C. Brown, Sébastien Incerti, Pierre Marin, Lydia Maigne, David Sarramia, Hervé Seznec, Vincent Breton

**Affiliations:** 1 Clermont Université, Université Blaise Pascal, CNRS/IN2P3, Laboratoire de Physique Corpusculaire, BP 10448, F-63000 Clermont-Ferrand, France; 2 Clermont Université, Université Blaise Pascal, Laboratoire Microorganismes Génome et Environnement, UMR CNRS 6023, BP 10448, F-63000 Clermont-Ferrand, France; 3 School of Mathematics and Physics, Queen’s University Belfast, Belfast, Northern Ireland, United Kingdom; 4 Université de Bordeaux, CENBG, UMR 5797, F-33170 Gradignan, France; 5 CNRS, IN2P3, CENBG, UMR 5797, F-33170 Gradignan, France; Mizoram University, INDIA

## Abstract

At very low radiation dose rates, the effects of energy depositions in cells by ionizing radiation is best understood stochastically, as ionizing particles deposit energy along tracks separated by distances often much larger than the size of cells. We present a thorough analysis of the stochastic impact of the natural radiative background on cells, focusing our attention on *E. coli* grown as part of a long term evolution experiment in both underground and surface laboratories. The chance per day that a particle track interacts with a cell in the surface laboratory was found to be 6 × 10^−5^ day^−1^, 100 times less than the expected daily mutation rate for *E. coli* under our experimental conditions. In order for the chance cells are hit to approach the mutation rate, a gamma background dose rate of 20 *μ*Gy hr^−1^ is predicted to be required.

## Introduction

When considering the impact of ionizing radiation on cellular systems from the environment, the absorbed radiation dose is considered by experimentalists. While this is appropriate in high dose regimes, it is less applicable in the growing field of low background biological research [1–3]. Absorbed dose measures a continuous energy deposition per unit mass, when in reality energy is deposited by ionizing particles along tracks. For low doses, these tracks do not always cross a significant proportion of cells in the populations studied in a biologically relevant time period [4]. The magnitude of this effect can be quantified using particle transport simulations to replicate biological experiments and their radiation environments.

This is significant for informing the interpretation of experiments carried out in very low background biological research, where since early experiments in the Pyrénées by Planel et al. [5, 6], it has been suggested that living systems respond to low radiation environments. A consistent feature of this early research was the appearance of inhibited growth of protozoa and cyanobacteria within the underground low background environment. Satta et al. [7] observed similar inhibitions arising in cells grown underground, when subjecting yeast to challenge experiments simultaneously in Rome and the Gran Sasso Underground Laboratory (a 6.7-fold difference in the absorbed dose). When cells grown for 120 generations in each environment were exposed to a challenging dose of a carcinogenic agent, higher fractions of recombinant and aberrant cells were found in the culture grown in the low background environment. In a recent study conducted at the Waste Isolation Pilot Plant in New Mexico, Castillo et al. [2] observed a reduction in the growth rates of both *Shewanella oneidensis* and *Deinococcus radiodurans* when they were grown for 48 hr in an extremely low background environment compared to a reference environment. Significantly, the bacteria were given no time to adapt to the radiation environment, and upon re-introduction to the reference environment, the bacteria immediately recovered their former rate of growth. Such a rapid response to the background is intriguing, suggesting that in a short amount of time living systems can ‘sense’ the level of radiation present. Despite these observations, simple estimations made for this experiment of the frequency with which radiation tracks strike bacteria place a strikingly small upper bound on the fraction of bacteria that could experience an ionization event in one generation [4]. Further work is needed to explain the mechanisms that permit a causal link between the radiation environment and an observable population-wide effect.

Beyond studying the relatively short-term impacts of a change in the radiative background when living systems are introduced to a low background environment, long term adaptive effects also present interesting results. Human TK6 cells grown simultaneously in the Gran Sasso laboratory and a reference radiation environment over six months by Carbone et al. [8] showed significantly different quantities of micronuclei formation after 1 min of exposure to a 2 Gy min^−1^ X-ray dose. Micronuclei formation, indicative of unrepaired chromatin damage, was greater in the cells cultured underground, suggesting that adaptation to the low background environment caused cells to lose some of their resistance to ionizing radiation. These differences in the radiation response of cells grown underground for long time periods provide a tantalising indication that cells adapt to their radiation environment.

While ionising radiation has traditionally been considered toxic at any level, experiments at low radiation backgrounds provide a window to explore the contention that responses to radiation dosage are hormetic, and that a small radiation dose may stimulate cells [9–11]. Interpreted in this light, past experiments in underground laboratories show results consistent with radiation hormesis, in line with experiments showing non-linear dose-response relationships in marine bacteria [12]. Indeed, a myriad of biological responses have been shown to exist in recent years that suggest low radiation dosages can have unexpected impacts on cells, of which the bystander effect is the most notable [13, 14]. Such responses, including genomic instability and transgenerational susceptibility to cancers (in large organisms) are possibly even mediated through epigenetic pathways [15]. The potential of a hormetic, adaptative response is strengthened by measurements made within the Chernobyl area, where haploid cells from birch pollen, and diploid cells from the seeds of evening primrose plants have shown an adaptation to the higher radiation levels now endemic in the region through improved DNA repair mechanisms [16].

In addition to adaptation, underground laboratories provide an environment where evolution can be studied while reducing the likelihood of radiation induced mutations. Genetic mutations arise from many factors native to the cell, such as translation and transcription errors [17], reactive oxygen species induced damage [18], and recombination of DNA in meiotic organisms. Ionizing radiation also contributes to genetic mutations both directly, through interactions between high energy particles and the DNA chain, and indirectly, through radiation-induced reactive oxygen species attacking DNA [19]. Isolating the independent effects of all these factors poses a significant experimental challenge: one way in which it may be approached is by replicating identical, controlled long term evolution experiments (LTEEs) in both low-background and ambient-background environments.

To this end, we are currently replicating the Lenski LTEE [20–22] in two different radiation environments, the Modane Underground Laboratory (LSM—Laboratoire Souterrain de Modane), located within the Fréjus road tunnel in the French Alps [23] and the Clermont-Ferrand Particle Physics Laboratory (LPC—Laboratoire de Physique Corpusculaire). The Lenski LTEE is the longest running controlled evolution experiment to date. It provides a framework well adapted for measuring the impact of ionizing radiation on the evolution of simple living systems. Here, twelve independent strains of *Escherichia coli* were grown in a nutrient limited medium over a 24 hour period. At the end of each 24 hour period, a small amount of the bacterial culture was pipetted into a new medium. The daily repair/growth/starvation cycle that occurred under these conditions provides a selection pressure that drives the evolution of the studied bacterial populations. From these independent parallel populations, a repeatable measurement of the mutation rate could be found.

Using simulation, the stochastic impact of the radiation background in biological experiments can be constrained, by calculating the frequency with which particle tracks intersect cells and deposit energy within them. Such simulations are of particular importance in informing the interpretation of low background biological experiments. Here the combined impacts of low dosages, small cell sizes, and relatively short experiment durations create a scenario where the interactions between radiation and cells need to be considered stochastically, rather than in terms of a dose absorbed (as has been widely used to date). Monte-Carlo based particle track simulation packages have seen wide use in simulating the impact of radiation upon cells in radiotherapy [24, 25] and are easily applicable to cellular dosimetry [26, 27]. Going further, Monte Carlo codes can simulate both direct and reactive oxygen species induced damage caused by radiation sources, both through explicit simulation [28] and analytical modeling of the chemical processes induced by radiation [29].

In this paper, we present a method by which the Geant4 simulation toolkit [30–32] can be used to accurately calculate the frequency with which ambient radiation sources interact with bacterial cells. We apply this method to our continuing LTEE in both the LSM and LPC. We show how many cells are impacted per unit time by the radioactive background, placing bounds on the maximum rate of mutations triggered by the ionizing background. More generally, these numbers are also interpreted in the light of short term low background experiments, giving a physical quantification of the extent to which bacterial cells may indeed be able to ‘sense’ the radiation present in their environment.

## Method

Given the impact of radiation on any system depends on its geometry, we initially present an overview of our experiment, as this influences the conditions under which cells experience the natural radiation environment. Next our simulation methodology is presented (The source code for our simulations is available through the Geant4-DNA website, http://geant4-dna.org [33]). We conduct simulations in two stages, first simulating the experimental environment to find the charged particles that may interact with cells before simulating the interactions between these secondary particles and individual cells. We call these the macroscopic and microscopic simulation levels respectively. In passing from the macroscopic to the microscopic level, we ensure that no information is lost. Finally we present the background sources relevant to our experiment and how they are each used as inputs to the simulations.

### Biological Conditions

In both the ambient radiation environment in the LPC and the low background environment provided by the LSM, we have been growing *E. coli* across multiple hundreds of generations. Bacteria are grown in 24 wells of a 96-well polypropylene microplate (Greiner Bio-One Item No. 780271), with each well containing 1 × 1 × 1.5 cm of Davis minimal broth [34] enriched with 250 mg L^−1^ glucose. Every day, the liquid culture was agitated constantly at 170 RPM at 37°C for 24 h. At the end of each 24 h cycle, 5 *μ*L of bacteria were transferred to a new microplate. Before the growth phase begins, the bacterial concentration was 1.7 × 10^6^ cells mL^−1^ which rose to 5.0 × 10^8^ cells mL^−1^. At the macroscopic level, the geometry of a 96-well microplate is considered, whereas at the microscopic level, a small repeating volume that models the final cellular density is simulated.

### Simulations at the Macroscopic Level

**Fig 1:**
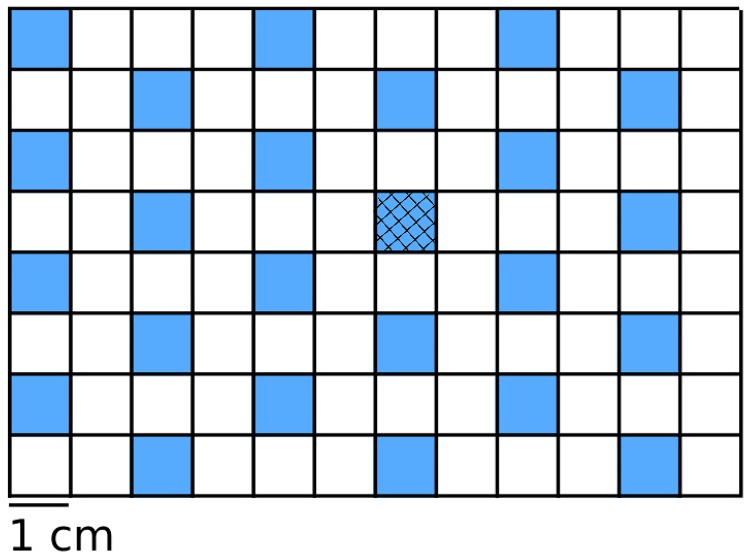
The 96-well microplate was simulated with 24 wells being filled with Davis minimal broth (blue). The polypropylene border to each well was 1 mm in width and 2 cm in depth. Liquid filled each well to a depth of 1.5 cm. A central well (hatched) was used as to measure dosages and secondary particles created.

Our first simulations consider the experimental environment. Within Geant4 (all simulations were made using Geant4.10.2.p01 MT) we modeled a 96-well microplate using polypropylene (Fig 1). The Davis minimal broth was modeled as water that was enriched elementally by the chemical composition of the Davis minimal broth (specifically matching the composition of Sigma-Aldrich product 15758), as the trace presence of these constituents, notably potassium caused a ≈10% increase in the neutron absorption cross section of the well [35]. The aim of this simulation was two-fold, we measured the dose deposited in the well in addition to recording the charge carriers created within the well, either directly from an internal source; from a charge carrying particle entering the well from the outside; or from physical processes where charged particles were created within the well by neutral particles that entered the sensitive volume (ie. photons and neutrons). By storing only charged particles created, and not the secondaries that these particles in turn created, we preserved the spatial correlations between all electrons created by ionization from any recorded charged particle within the sensitive volume, as they were ‘re-created’ at the microscopic level. Particles were still tracked after they were saved in order to measure the energy that they deposited in the well and to observe whether secondary particles were later created that re-entered the well even after a given particle had left. When particles were saved, their positions, momentum directions, species’ and energies were recorded. At the conclusion of the run, these were placed into a phase space file. This phase space file served as the input for microscopic level simulations.

### Simulations at the Microscopic Level

We model the microscopic level as a much smaller cube of side length 200 *μ*m filled with 4000 bacterial cells placed randomly in the domain. Each cell was modeled as a rod, defined by a cylinder of radius 0.5 *μ*m and length 2 *μ*m, capped at each end by a radius 0.5 *μ*m hemisphere [36].

**Fig 2:**
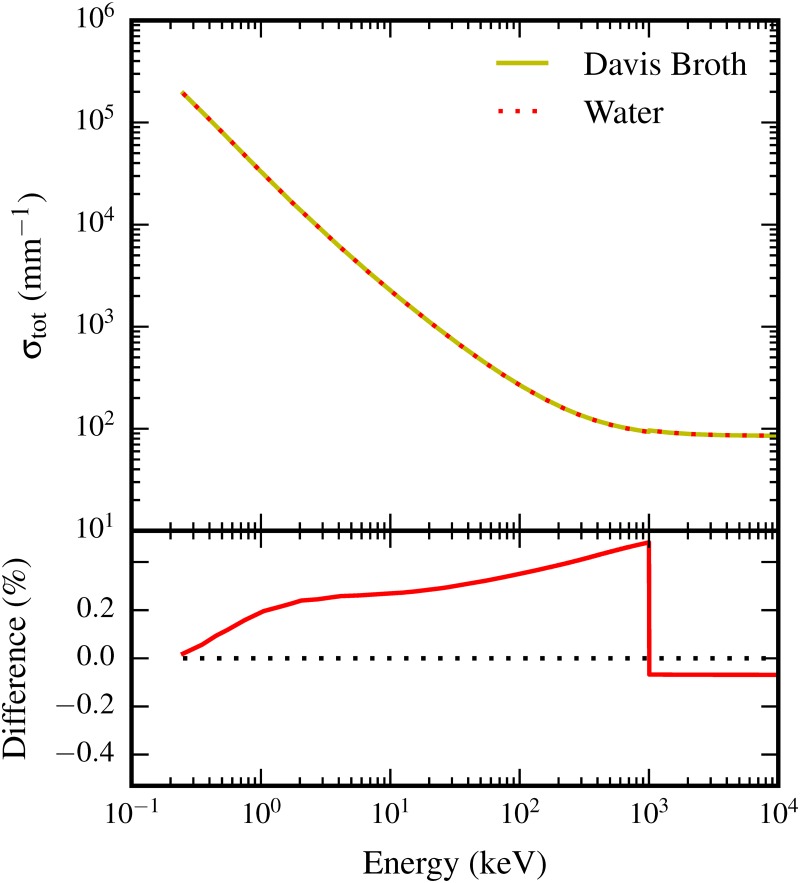
The top panel shows the total electron interaction cross section (*σ*_tot_, given per volume, for *ρ* = 1 g cm^3^ solutions), which determines the likelihood of electron interactions occurring for a given electron energy, in both water and Davis broth. The difference between these curves is shown in the bottom panel. Across the range of electron energies considered in our simulations, water approximates the Davis broth solution to within < 0.5%.

Unlike at the macroscopic level, the cells were simulated as being made of high density water in a water medium. The density inside cells was set at 1.10 g cm^−3^ [37, 38]. Approximating the elemental composition of the cells as water makes little difference at this level, given electrons dominate the input spectrum, and their cross sections for scattering, bremsstrahlung and ionization are not strongly influenced by the presence of elements found at low concentrations in biological media. Specifically, for equal densities, the total electron interaction cross section for these processes obtained using Geant4 from the Penelope model set differs by less than 0.5% between a Davis minimal broth solution and water (Fig 2), which is less significant than the variation in simulation results coming from the choice of model used.

**Fig 3:**
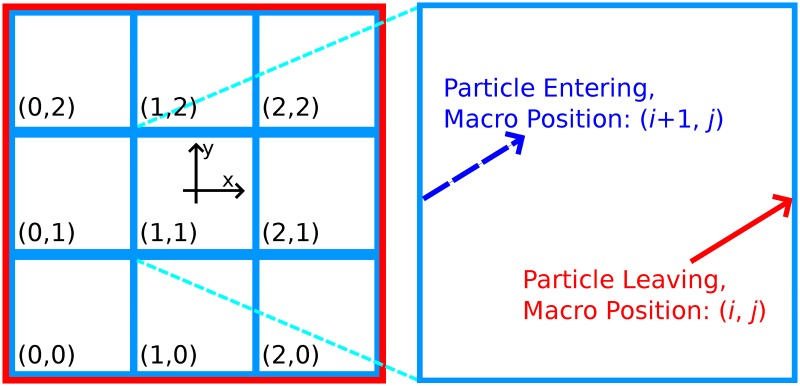
The repeating boundary condition allows the simulation area at the macroscopic level to be broken up into a series of microdomains with (*x*, *y*) ∈ [−100 *μ*m, 100 *μ*m]^2^ (left). Each microdomain contains the same geometry due to the repeating boundary, however each track stores the identity of its current macroscopic domain, here labeled in the bottom right of each small cube. When a particle leaves a microdomain (right), it re-enters at the other side, due to the periodic boundary. When this occurs, the track updates its stored position in the macroscopic view.

While the simulated domain is significantly smaller than a well in a microplate, and would be easily escaped by even low energy electrons, given 100 keV electrons have a mean penetration distance of 140 *μ*m in water [39], we prevented this by enforcing a periodic boundary condition (Fig 3). This caused particles leaving the domain to loop around and enter at the other side. So that particles may still leave the simulation after traveling the length of the well, particles were created with a set of ‘global’ co-ordinates that in combination with the local co-ordinates inside the 200 *μ*m box allowed the particle to be localized within a virtual well (the virtual well is equivalent to tessellating the real well with many 200 *μ*m cubes). Whenever a particle crossed the periodic boundary its global co-ordinates were updated, and the particle was killed when the global coordinates corresponded to a point outside the well.

Primary particles were created in the microscopic domain with positions, direction vectors and energies specified in the phase space file created at the macroscopic level. They are assigned both global and local co-ordinates based upon the position read from the phase space file. When particles entered cells, the energy they deposited in each cell was saved, as well as their position, direction and energy at entry. The number of cells hit in each event was also saved.

### Background sources and simulation parameters

Previously, we discussed the relative contributions of different background sources to biological experiments and presented measured dosages pertinent to these experiments in both above and below ground environments [40]. Briefly, these sources are the terrestrial gamma background, the cosmic charged and neutron background and radiation introduced to the experiment from potassium added to the bacterial growth culture. While airborne radon is a major contributor to the background radiation dose in humans, our experimental geometry significantly reduces the exposure of cells to airborne radon at typical concentrations, hence we do not consider it here. Additionally, given the small quantity of water in each well, the radon concentration in each well is limited to around one decay per day.

Two environments are considered within this work. These are the LPC which provides a reference surface-level radiation environment for our experiments, and the LSM as an underground environment. At the LPC, each form of background radiation mentioned previously is relevant to the experiment. The LSM’s position underground shelters it from the majority of cosmic rays: here the only significant sources of radiation are the terrestrial gamma background and the background from the nutritive medium.

The same physics models were used for each source configuration. At both simulation scales hadronic processes were modeled using the QGSP_BIC_HP physics list and the ‘Option 4’ standard EM physics list was used to simulate EM processes with a low energy production threshold of 100 eV. At the macroscopic level a secondary production cut of 1 *μ*m was used, and at the microscopic level the secondary cut was reduced to 10 nm. We also set the maximum step size to 10 nm for these simulations.

Using a Monte Carlo particle transport simulation at such small length scales, we were concerned that some continuous multiple scattering models employed could introduce errors into our analysis. To better understand the impact of our choice of physics models on the final simulation results, we simulated the transport of 10^6^ 200 keV electrons in the same geometry as the microscopic simulation, with the only change being that the repeating region continued indefinitely. In addition to our simulation results, we compared the interactions of these electrons with cells using the standard electromagnetic Option 4 physics constructor, as well as the low energy electromagnetic Penelope, Livermore and Geant4-DNA option 2 physics constructors [41]. Notably, the Geant4-DNA Physics models [42] provide fully discretized low energy electromagnetic processes. This allows us to quantify the extent to which the approximation of scattering as a continuous process impacts our results.

#### The gamma background

We have based our simulations of the terrestrial gamma background upon measurements made within the LSM using a high purity Germanium spectrometer [43], and our own measurements of the gamma spectrum both in the LSM and at the LPC, made using a Thallium-doped Sodium Iodide handheld gamma spectrometer. The Ge measurements found a background gamma flux in the center of the great hall of 0.301 *γ* cm^2^ s^−1^ across all considered flux bins. As our measurements indicated that the gamma spectrum in the LSM is close to that measured in Clermont-Ferrand [40], surface measurements can be obtained by scaling the results found using the underground spectrum by 6.4 (thus ensuring a surface dose of 150 nGy hr^−1^, consistent with measured values). At the macroscopic level, the energy binned gamma fluxes were simulated as isotropic, by considering the source to be an *r* = 3 cm gamma-emitting sphere around a central well in the microplate, which was chosen to be the detector. Incoming gamma rays create electrons by the Compton and photoelectric effects, as well as occasional positrons by pair production. The positions, directions and energies of these secondary particles created in the chosen well was then used to seed the simulation at the microscopic level. 10^8^ primary gamma rays were simulated at the macroscopic level, equivalent to 10.8 days of exposure in the LSM (after scaling, this is 1.68 days in the LPC). At the microscopic level, 2 × 10^6^ secondaries were simulated, randomly selected from those created at the macroscopic level.

#### The nutritive background—Potassium-40

The radiation background from the nutritive medium in biological experiments is dominated by the contribution from *β*^−^ emission by ^40^K, which dominates the absorbed dose from ^40^K *γ* emission and ^14^C *β*-decay by over two orders of magnitude. To model the secondaries that enter each well in our experiment, we chose a central water-filled well in the microplate geometry to be the sensitive region, and defined that well and its six closest filled neighbors to be sources. Within each of these seven source wells, electrons were created with a uniformly random distributions of position and emission direction. The energy spectrum was defined by the *β* spectrum of ^40^K [44]. 10^7^ events were simulated at the macroscopic level, corresponding to 105 days of real time. At the microscopic level, 10^6^ events were simulated, drawn randomly from the phase space file created at the macroscopic stage. For this source, the phase space file consists solely of electrons with an energy spectrum very similar to that at the macroscopic level, as all electrons created within the sensitive region are saved as soon as they are created.

#### The cosmic neutron background

The neutron background was simulated using an algebraic expression for the neutron differential flux in New York [45] between 100 keV and 500 MeV. Within this range, the differential flux is *ϕ*_*n*_ = 5.96 × 10^−3^ cm^−2^ s^−1^. In simulation, this source was modeled as a disc of radius 10 cm situated 30 mm above the center of the microplate emitting neutrons uniformly along its surface with an isotropic angular distribution. This causes the particles to arrive at the well with an angular distribution of cos^2^ *θ* where *θ* is the angle to the vertical. Neutron interactions within the well create primarily free protons, electrons, alpha particles and ^16^O ions, while many other ions are created in small quantities. Given the high kinetic energy of the incident neutrons, these particles often also have a high kinetic energy, sometimes a few hundred MeV’s. All these particles are saved at the end of the simulation, to be read into the microscopic simulation. At the macroscopic level, 10^8^ particles were simulated corresponding to 98.4 days of real time. 10^6^ particles were then simulated at the microscopic level, drawing randomly from the list of particles created at the macroscopic level.

#### The cosmic muon background

We simulated the cosmic muon background based upon the spectrum predicted by Chirkin [46], which provides the differential muon flux as a function of both muon momentum and direction. While this derivation is for muons with momenta above 600 GeV/c it models low energy to an acceptable level of accuracy for our simulations. We considered muons with momenta between 0.1 − 50 GeV/c, and simulated 54% of muons as *μ*^+^, with the rest as *μ*^−^. The total integrated flux within this range was 3.4 × 10^−2^ *μ* cm^−2^ s^−1^, in reasonable agreement with the accepted sea level muon flux [47]. We simulated 10^8^ *μ* as the primary source at the macroscopic level, created at random positions in an *r* = 15 cm disc positioned 3 cm above the microplate, with emission angles based upon the integrated differential flux formula. This is normalized to 48.7 days of real time. At the microscopic level, 10^6^ particles generated at the macroscopic level were randomly drawn and tracked to measure the interactions between muons and electrons from muon-disintegrations and bacterial cells. The energy of these particles spanned the same range as the input muon spectrum, given that the energy loss of muons traveling through air is small.

## Results

As the choice of physics models used in any simulation impacts the outcome, we first present our brief comparison of physics models for the microscopic level simulation. The distribution of energy depositions in cells and the number of cells that had energy deposited in them are summarized in Table 1. The spectrum of energy depositions follows a Landau distribution as it is effectively a sampling of the energy deposited by a decelerating charged particle. Accordingly, we present percentiles of this distribution rather than a mean, as the mean carries little meaning for this type of distribution. Between each model, the distributions of energy deposition are significantly different (a Kolmogorov-Smirnov test between any two models shows the distributions are dissimilar to >5*σ*), however the parameters relevant to our study show broad agreement between models. The number of cells hit in the simulation (those experiencing at least one energy deposit) agrees within 10% between models, and the measures of energy deposited, while being different distributions, differ by ≈ 10% beyond the 50th percentile. These two observations allow us to approximate the errors in our results coming from the physics models chosen to ≈ 10%.

**Table 1:** Variation between simulation outputs for different physics models. Energy deposits are given at the 25th, 50th and 75th percentile of the distribution.

EM Physics Constructor	Cells hit	*E*_dep_ (eV)		
		25th	50th	75th
Geant4-DNA Option 2	541568	108	212	426
Standard Option 4	534138	152	218	414
Penelope	531943	125	222	446
Livermore	570801	114	191	396

**Fig 4:**
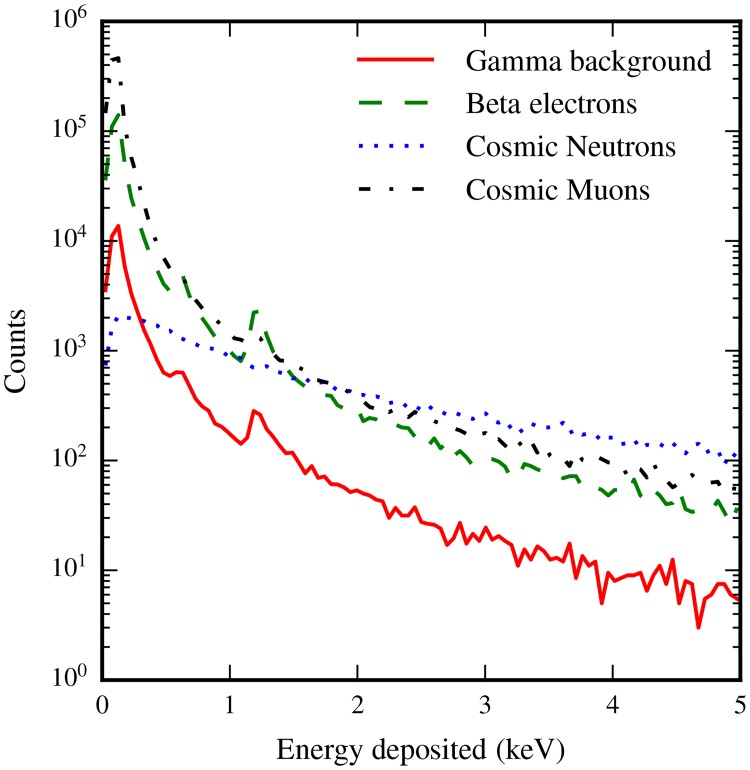
Each source deposits energy in cells according to different Landau-like distributions. Energy depositions are normalized to the hit rate, indicating for each source the chance a specific amount of energy is deposited in it in a day. The peaks near 600 eV and 1.2 keV in the *γ*-background and *β*-electron spectra are related to the emission of one or two short-traveling Auger electrons emitted by Oxygen atoms within cells, in addition to the energy deposited by other processes.

Aided by this preliminary study which quantifies the precision of our simulation results, we turn our attention to our measurements of energy deposition in cells from different background sources. Table 2 indicates the frequency with which cells are subjected to a radiation induced energy deposition for each source we considered. The hit frequency is normalized by the total number of cells considered in the study, giving a quantity that corresponds to hits per cell per day, or alternatively, the chance that any given cell is hit in a one day period. The specific distribution of energies deposited in a cell per day is shown in Fig 4.

**Table 2:** Frequency with which ionizing radiation from background sources interacts with *E. coli* cells, the median and modal energies deposited in each interaction. These quantities are correspond to the surface environment at LPC.

Source	Dose Rate	Hit frequency	*E*_dep, median_	*E*_dep, mode_
	(nGy hr^−1^)	(day^−1^ cell^−1^)	(eV)	(eV)
*γ* background	150	3.6 × 10^−5^	140	100
^40^K *β*-decay	26	8.2 × 10^−6^	120	120
Cosmic *μ*	45	1.6 × 10^−5^	110	120
Cosmic *n*	4.4	1.4 × 10^−7^	1.2 × 10^3^	670
Total	225	6.0 × 10^−5^	-	-

Studying just the dosages found using the inputs available, it is evident that the gamma background dose is elevated when compared to the standard population weighted average dose of 60 nGy hr^−1^ [48]. This is caused by naturally higher radiation levels at the LPC due to the soil composition. Similarly, the muon dose is 27% higher than modeled values would predict for a site at our elevation 400 m), where the predicted dose is 33 nGy hr^−1^ [49].

The total number of interactions between an *E. coli* cell per day and the radiation background at the surface is 6.0 × 10^−5^ day^−1^, indicating that on average roughly 1 in 20,000 would be expected to interact with ionizing particles from the radiation background on a given day. Underground, the 6.4-fold reduction in the gamma background reduces the frequency of interactions per day for a given cell to 1.37 × 10^−5^ day^−1^. Suppressing the gamma background entirely leaves only the contribution of ^40^K, giving a 7.3-fold reduction in the cellular hit rate compared to the background of 8.2 × 10^−6^ day^−1^.

**Fig 5:**
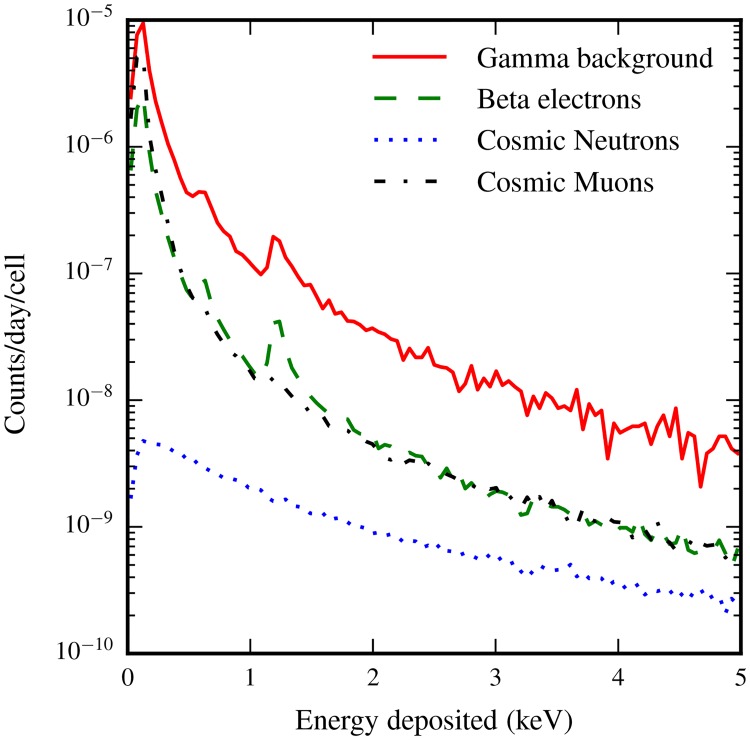
When the energy depositions are normalized to 10^6^ primary events, the characteristics of each source become clearer. Sources that travel further through the medium impact more cells, whilst the significantly higher LET from neutron-induced ions is reflected in the flatter distribution of energy deposits from this source.

To better understand the nature of energy deposition induced by each source, in Fig 5 we show the distribution of energy deposits per 10^6^ simulation events. Energy depositions correspond with what one would expect based on the particle transport characteristics of each input source, that is to say that higher LET sources deposit energy according to a flatter Landau distribution, whilst the exact quantity of cells hit is determined by the mean distance particles would travel through the water medium simulated.

Comparing the backgrounds from the gamma background and the nutritive medium, for the same input dose, electrons from beta decay of potassium in the nutritive Davis minimal broth impact more cells, albeit with a lower median energy deposition (Table 3). This is consistent with the expected comportment of electrons: higher energy electrons will have longer paths relative to their energy given linear energy transfer is inversely proportional to a particle’s energy, and the mean beta electron energy from ^40^K is 511 keV in comparison to 74 keV for electrons created from the gamma background. Secondaries created from the neutron background, being predominately ions, tend to have very high LET values leading to both a large energy deposit when cells are hit, and a relatively small quantity of cells hit given the short path neutrons traverse.

**Table 3:** Expected radiation interactions in one *E. coli* cell per unit dose from natural background sources. These quantities are sensitive to both the geometry of the cell considered and the experimental geometry.

Source	Expected interactions	Mean source energy
	(nGy^−1^)	(keV)
*γ* background	1.0 × 10^−8^	7.4 × 10^−2^
^40^K *β*-decay	1.3 × 10^−8^	5.11 × 10^−1^
Cosmic *μ*	1.3 × 10^−8^	1.34 × 10^7^
Cosmic *n*	1.3 × 10^−9^	1.1 × 10^4^

It is interesting to note that both tracks from ^40^K beta decay electrons and cosmic muons traverse the same amount of cells per unit dose of radiation. Additionally, the spectrum of energy depositions from muons strongly resembles that caused by beta decay electrons. This is tied to the similar LETs of 511 keV electrons (2.04 MeV cm^−1^, c.f. [39]) and muons (2.55 MeV cm^−1^ for 14 GeV muons, c.f. [50]), however based upon this alone muons ought to interact with comparatively fewer cells per unit dose deposited in the material. The additional component in the muon interactions comes in part from a contribution to the interactions made by electrons that enter the well as the children of decaying muons.

## Discussion

By conducting these simulations, we have sought to quantify the impact of background radiation in biological experiments, and in doing so, guide the interpretation of the growing body of low background biological experiments. Measurements of the frequency with which the radiative background interacts with cells provides an upper bound for the size of most radiation induced effects. Such effects are not limited to genetic damage induced by radiation, as radiation may also affect cells and induce death by damaging proteins directly and through oxidation [51, 52].

Physically, the spectrum of energy depositions from each source (Figs 4 and 5) reveals a significant amount of information about the nature of the energy being deposited within cells. Clearly evident in the spectra are peaks related to the Auger emission spectrum of water (near 500eV), emphasizing the significance of this process in low energy cellular dosimetry. This is especially important considering the otherwise low energy deposits that occur in the absence of Auger effects. For lepton backgrounds, the modal and median energy deposits are near 100 eV per cell hit, meaning that the emission of just one Auger electron in a cell has the capacity to significantly affect the energy that would otherwise be deposited. It is worthwhile to give some consideration to the impact of these energy deposits in terms of the volume they impact through the radiolysis of water. In the domain we are interested in, a majority of the electrons energy is deposited in water as ‘spurs’ along the electron’s path. Spurs are bead-like regions where a 40 − 100 eV energy deposit ionizes and excites water molecules, which react and diffuse in a cloud with a diameter of 4 nm [19]. Considering that the median energy deposit from lepton sources is at its highest 140 eV, the majority of cells impacted by radiation contain only one to three such regions, emission of a single Auger electron, which has a maximum track length of 11 nm [53] significantly contributes to the energy deposited, and also to the volume within the cell impacted by radiation. More importantly perhaps, this energy is deposited over a well-localized region in space, while spurs can be separated by several hundred nanometers. It would be interesting to pursue simulations further to quantify the precise impact of this on cellular structures.

As mentioned in the introduction, these simulations were conceived around an ongoing long term evolution experiment in the LSM. Between the reference radiation environment at the LPC and the reduced radiation environment at the LSM, cell cultures are grown experiencing either 6.0 × 10^−5^ interactions cell^−1^ day^−1^ or 8.2 × 10^−6^ interactions cell^−1^ day^−1^. From these figures, we seek to determine whether the evolutionary behavior of *E. coli* ought to change between these regimes. Such an evaluation is comparatively simple: the upper bound on the point mutation rate of *E. coli* across the first 20,000 generations of a long term evolution experiment is 7.4 × 10^−4^ mutations per generation [54]. Given we grow 8.23 bacterial generations per day, the upper bound on the point mutation rate in our experiments is 6.1 × 10^−3^ mutations per day: 102 times higher than the frequency with which radiation interacts with cells. The significance of this comparison indicates that, following the assumption that radiation should not produce mutations that differ significantly in their effects to those of biological processes, in an *E. coli*-focused long term evolution experiment, the radiation background should not significantly affect the evolutionary behavior of the population, due to the relative infrequence with which it impacts bacterial cells at the surface radiation level. With the caveat that a comparison between the daily mutation rate and radiation interactions per day is a comparison of two upper bounds, the evolutionary behavior of bacterial cells should not be significantly different in an underground environment compared to a surface-level laboratory, as the impact of the radiation background is less than 1% of that from biological processes.

The independence of radiation induced interactions, and thus, possible radiation induced mutations from the point mutation rate is not surprising. The mutation rate is a biological parameter that is subject to selection, which is optimized according to the dynamics of the host population rather than by a uniform oxidative stress. Typically, its value is limited by random genetic drift: the variation in the frequency of different alleles within a population that comes from random sampling of a population. This limit arises because while proteins could theoretically be synthesized in the cell to reduce the mutation rate, after a point this becomes disadvantageous when the cost to the organism of having such proteins does not significantly outweigh the gains from reducing the total amount of genetic variation between generations [55]. Certain situations may also favor the appearance of vastly higher mutation rates, as is often marked by the appearance of mutator alleles in evolution experiments [56, 57]. The existence of radiation-tolerant bacteria such as *D. radiodurans*, and experiments forcing the evolution of radio-resistance in *E. coli* [58] indicate that when oxidative stresses are considerable, species evolve mechanisms to protect themselves from oxidative damage. In many ways this is both a by-product of the cell evolving mechanisms that allow it to survive oxidative stress as well as the cell attempting to select a mutation rate that is optimal for its environment, as each of these goals are mutually compatible. Nevertheless, there remains no significant reason why such a mutation rate should be particularly correlated with the radiation environment given the other forces involved in selection.

Exploring these responses in the context of controlled increases in the background radiation does present further avenues of future study. Long term evolution experiments show that a three-fold increase in the mutation rate caused by transfecting cells with a mutator gene can produce observable changes in the fitness trajectory [59]. Simulations such as those performed here can be used to determine which radioactive sources best increase the quantity of cells impacted by radiation so that this rate approaches or exceeds the mutation rate. For the sources considered here, an increase in the background rate to ∼20 *μ*Gy hr^−1^ would be sufficient to cause the rate at which cells are impacted by radiation to be near equivalent to the point mutation rate. Whether this increased radiation level would favor mutations linked to radioprotection rather than the fitness experiment itself needs careful evaluation. One study from the Chernobyl environment showed that background absorbed dose rates of up to 75 *μ*Gy hr^−1^ do not seem to encourage the formation of radio-resistant sub-strains [60], however a more recent study showed that resistance to *γ*-radiation was augmented in bacteria living in bird feathers that grew in radiation environments only a few times above the standard background (450 nGy hr^−1^), compared to bacteria found in feathers at both standard and significantly elevated (2.9 *μ*Gy hr^−1^) backgrounds [61]. Controlled, long term low-dose evolution experiments could even elucidate whether different radioprotective mechanisms evolve in different radiation environments.

There remains scope for the possibility that radiation may interact with biological systems in ways that conflict strongly with the assumptions made in the preceding paragraphs. Much as the likelihood of one type of mutation may become more or less likely depending on the genome of a cell (e.g. the *mutY* allele increases G:C to T:A transversions [62]), one could propose the idea that radiation could act as a trigger for less likely mutations. Measuring and quantifying this would be challenging, however this does leave a mechanism by which the radiation background could impact the evolutionary behavior of a population. Our measurements of competitive fitness in different radiation environments are still ongoing, and may shine further light on this possibility when completed. Even if the evolutionary behavior of a cell population shows no dependence on the radiation environment in the first thousand generations of an LTEE however, this does not eliminate the potential for radiation to play a subtler, longer term role in LTEEs. As the cell population becomes increasingly well adapted to its environment, measurements at much later generation times could potentially show a fitness dependence on radiation environment were radiation responsible for rare mutations, as the supply of non-radiation induced mutations could become exhausted. Whether this is possible is debatable, given that even after growing 50,000 generations of *E. coli*, the measured fitness of the bacteria continues to grow seemingly without bound [22].

Cast in the light of other low background experiments, the relatively low frequency of interactions between the radiation background and cells challenges existing assumptions about the mechanisms by which bacterial cells have seemed to ‘sense’, in a relatively short amount of time (days up to a week), that they have been transferred to a low background environment. In the introduction, we presented the Castillo et al.’s measurement of impaired bacterial growth in a low background environment compared to a reference environment after just 24 hours growth with the radiation background suppressed [2]. Repeating our simulations for the experimental and cellular geometries used in their experiment (where *D. radiodurans* cells were simulated as spheres of radius *r* = 1.5 *μ*m exposed to 71.3 nGy hr^−1^ from an isotropic 1.46 MeV *γ*-ray source, and a 7.2 nGy hr^−1^ exposure from internal ^40^K *β*^−^-decay), we estimate that the chance a radiation track deposits energy in a *D. radiodurans* cell in a day is 1.3 × 10^−4^, significantly lower than the upper bound on this figure given by Katz [4], largely due to our consideration of track structures. Note that despite the lower dose in this experiment as compared to our *E. coli* simulations, there are more interactions per day due to the larger size of *D. radiodurans* cells compared to *E. coli*, and the higher LET of secondary electrons induced from the *γ*-background used by Castillo et al., compared to a standard terrestrial background spectrum. Another way of interpreting these figures is then to say that Castillo et al. notice a population wide effect when only ≈ 0.01% of the cell population is actually able to notice a decrease in the radiative background in a one day long period. While bacterial cells can communicate, for example through the secretion of outer membrane vesicles [63], the emergence of a population wide effect coming from such a small fraction of the cell population is startling and warrants further investigation. While the decrease in growth rate is consistent with the hypothesis that the radiation dose response is hormetic, the speed with which the change occurs remains to be explained.

Beyond considering the rapidity with which a population level change has been reported to occur in a low background environment, it’s worth also noting that the time scale on which cells interact with the background is significantly longer than their division time. Assuming for a cell that the likelihood of interacting with the radiation background scales with its surface area, an animal cell (*r* ≈ 15 *μ*m) is hit 900 times more often than an *E. coli* cell (*r* ≈ 0.5 *μ*m). Thus even larger cells are still hit relatively rarely (on average, once every 25 days). In long duration experiments across both yeast and mammalian cells, evidence of a hormetic response to radiation has been observed in low background experiments [3, 7, 8, 64], where a small level of radiation seems to stimulate oxidative response mechanisms. Yet the mechanism by which information about very infrequent radiation energy deposition events is passed through the cell population or down the cell lineage remains to be understood.

This issue can also be further explored in simulation. Little work has been done simulating at a genome level the impact of radiation on bacterial systems. Whilst the frequency of interactions between a radiation source and a cell provides useful upper bounds on the amount of damage that could be incurred, we are currently unable to clarify the nature of this damage. Given an appropriate geometrical description of a cell, detailed simulations where both physics and radio-chemistry are simulated to high precision can enable the quantification of single and double strand breaks of DNA from radiation sources. Importantly, these simulations we have presented provide a means of obtaining the input spectrum of ionizing particles that interact with cells from an environmental source. Beyond this, simulations of proteins in addition to DNA could enable a clearer understanding of the role of protein damage in cell death by radiation. Of course, comparisons between simulation track structure codes and biological experiments are important in validating the simulation codes used.

## Conclusion

We have presented a method for better understanding the impact of low radiation doses on a population of individual cells. While very low radiation background biological experiments describe the natural radiation background as a stress that they seek to eliminate or reduce, here we have shown this stress affects less than 0.01% of the cell population per day in a model bacterial system. This conclusion is difficult to reach when an experimentalist only considers a reduction in the absorbed dose between environments. Despite this, underground biological experiments to date have shown that cells grown in underground environments have responded to their environment, sometimes very rapidly.

As this study accompanies a long term evolution experiment being conducted in an underground laboratory, we have found it useful to compare the maximum impact of radiation on the *E. coli* genome to observed mutation rates, and have shown that biological effects likely have a 100-fold stronger impact on mutations than radiation could. Work is required however to quantify the precise nature of radiation induced genetic effects as they may differ in unexpected ways from damage sustained from biological sources.

We’ve shown that detailed track structure simulations can quantify the stochastic impact of radiation on living systems at the background level. While radiation dose may seem to indicate a uniform deposition of energy in a volume, the fundamental mechanisms by which this energy is deposited are not continuous, an effect that becomes very noticeable at low dosages, and at small volumes. Both these conditions are met in underground experiments, and both need to be addressed in interpreting the results of very low background radiation experiments.
